# Concentration dataset for 4 essential and 5 non-essential elements in fish collected in Arctic and sub-Arctic territories of the Nenets Autonomous and Arkhangelsk regions of Russia

**DOI:** 10.1016/j.dib.2019.104631

**Published:** 2019-10-18

**Authors:** Nikita Sobolev, Evert Nieboer, Andrey Aksenov, Tatiana Sorokina, Valery Chashchin, Dag G. Ellingsen, Yulia Varakina, Elena Plakhina, Dmitry Kotsur, Anna Kosheleva, Yngvar Thomassen

**Affiliations:** a Northern (Arctic) Federal University named after M. V. Lomonosov, Arctic Biomonitoring Laboratory, Severnaya Dvina Emb. 17, 163002, Arkhangelsk, Russia; b Department of Biochemistry and Biomedical Sciences, McMaster University, Hamilton, ON, L8S 4K1, Canada; c Northwest Public Health Research Centre, 2-Sovetskaya str. 4, 191036, St. Petersburg, Russia; d National Institute of Occupational Health, P.O. Box 5330, Majorstua, N-0304, Oslo, Norway; e Norwegian University of Life Sciences, N-1432, Ås, Norway; f National Research University Higher School of Economics, Myasnitskaya str. 20, 101000, Moscow, Russia

**Keywords:** Essential and toxic elements, Indigenous people, Russian Arctic

## Abstract

The raw concentration data for the research article entitled “Essential and non-essential trace elements in fish consumed by indigenous peoples of the European Russian Arctic” (Sobolev et al., 2019) [1] are herein presented. Fifteen fish species were collected in the Nenets Autonomous and Arkhangelsk Regions of the Russian Federation and were analysed for 9 elements (As, Cd, Co, Cu, Hg, Ni, Pb, Se and Zn). The sampling sites were located in the European parts of the Russian Arctic and sub-Arctic territories. Within these territories, Nenets indigenous peoples commonly catch and consume local fish. Based on questionnaire data, local fish sources constituted ∼ 90% of the total fish consumed by endemic individuals living in these regions. The data summarized in this publication fill a gap in knowledge.

**Table:** Specifications Table

Subject	Environmental Science (General)
Specific subject area	Intake of essential and toxic elements from locally harvested fish
Type of data	Tables and charts
How data were acquired	Questionnaire and inductively coupled plasma mass-spectrometry (ICP-MS) Aurora Elite (Bruker Daltonik GmbH, Bremen, Germany)
Data format	Raw and analysed data
Parameters for data collection	The researchers bought fish from local indigenous fishermen that were frozen immediately at −20 °C, refrigerated and then transported to Arkhangelsk. A detailed questionnaire was administered to a local indigenous population in 2017–2018
Description of data collection	Homogenized freeze-dried fish muscles were digested by 5 ml of concentrated nitic acid using a hot-block system at 105 °C, and were subsequently analysed by ICP-MS
Data source locations	Indiga, Nenets Autonomous region, Russia. Indiga (67.65–67.71 N 48.75–49.03E); Krasnoe, Nenets Autonomous region, Russia. Pechora 1 (67.97–68.03 N 53.96–54.01E); Nelmin-Nos, Nenets Autonomous region, Russia. Pechora 2 (67.93 N 52.96E); Ustie, Nenets Autonomous region, Russia. Pechora 3 (67.56 N 52.53E); Kuloi village, Arkhangelsk region, Russia. Kuloi 1 (64.97 N 43.50E) and Kuloi river, Arkhangelsk region, Russia. Kuloi 2 (65.97 N 43.49E). Samples were analysed at the Northern Arctic Federal University named after M.V. Lomonosov, Arctic biomonitoring laboratory, Arkhangelsk, Russian Federation
Data accessibility	Data are available in the current publication and have also been placed in a public repository: “Data for 4 essential and 5 non-essential elements in fish collected in Arctic and sub-Arctic territories of the Nenets Autonomous and Arkhangelsk Regions of Russia” Direct URL to data: https://doi.org/10.17632/schjsjfn3x.1
Related research article	Author names: Nikita Sobolev, Andrey Aksenov, Tatiana Sorokina, Valery Chashchin, Dag G. Ellingsen, Evert Nieboer, Yulia Varakina, Elena Veselkina, Dmitry Kotsur and Yngvar Thomassen. Title: Essential and non-essential trace elements in fish consumed by indigenous peoples of the European Russian Arctic Journal: Environmental Pollution DOI: https://doi.org/10.1016/j.envpol.2019.07.072

**Table:** 

**Value of the Data** • The comprehensive raw data set presented has not been reported previously. • These data will be helpful for researchers involved in nutritional and general health assessments and related research. • The data also help to identify potential dietary sources of essential and non-essential elements for indigenous communities in the European Russian Arctic/Subarctic. • Our findings supplement those of pan-Arctic biomonitoring studies, and are suitable for inclusion in pertinent reports/overviews.

## 1 Data

**Fig. 1:**
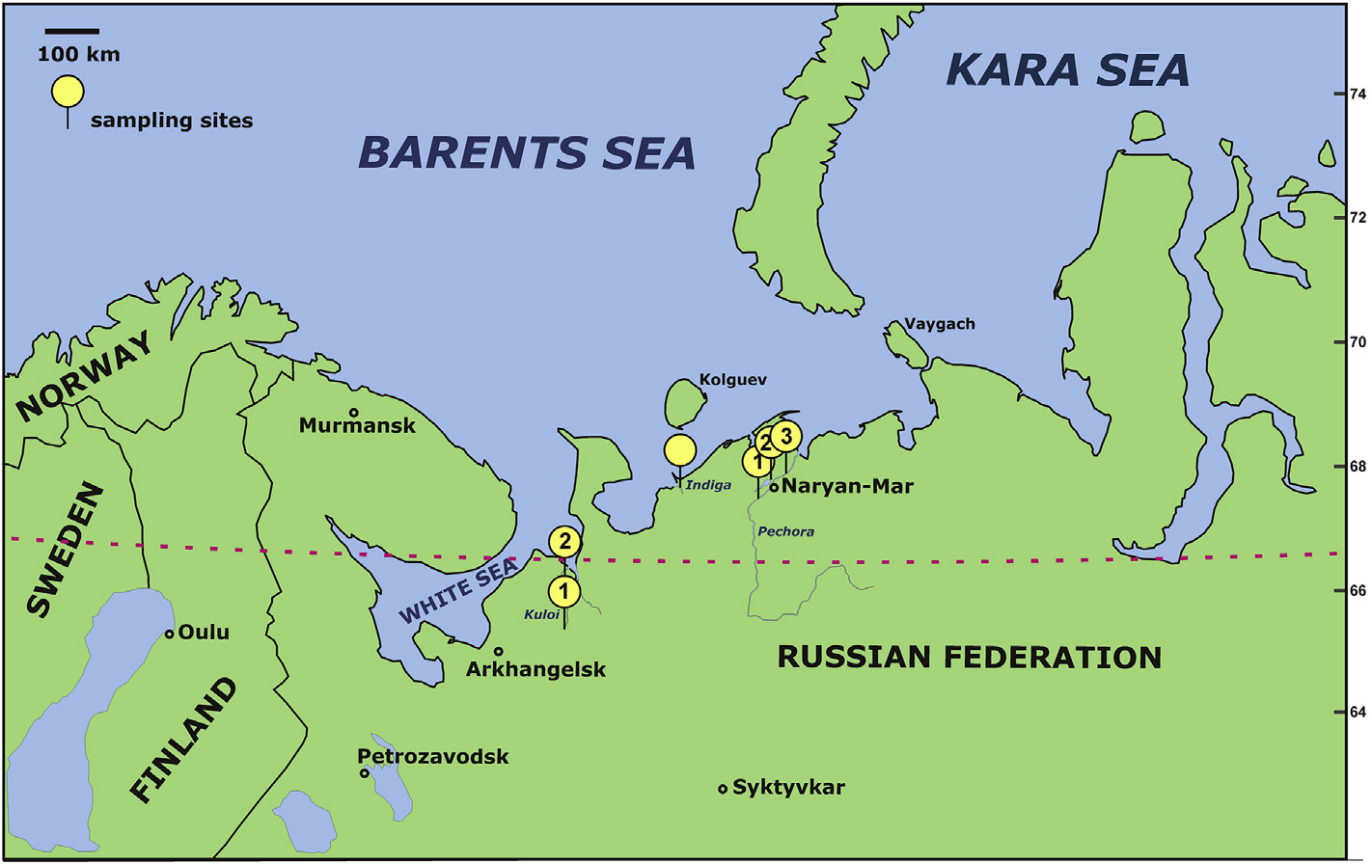
Map showing the fishing sites.

**Fig. 2:**
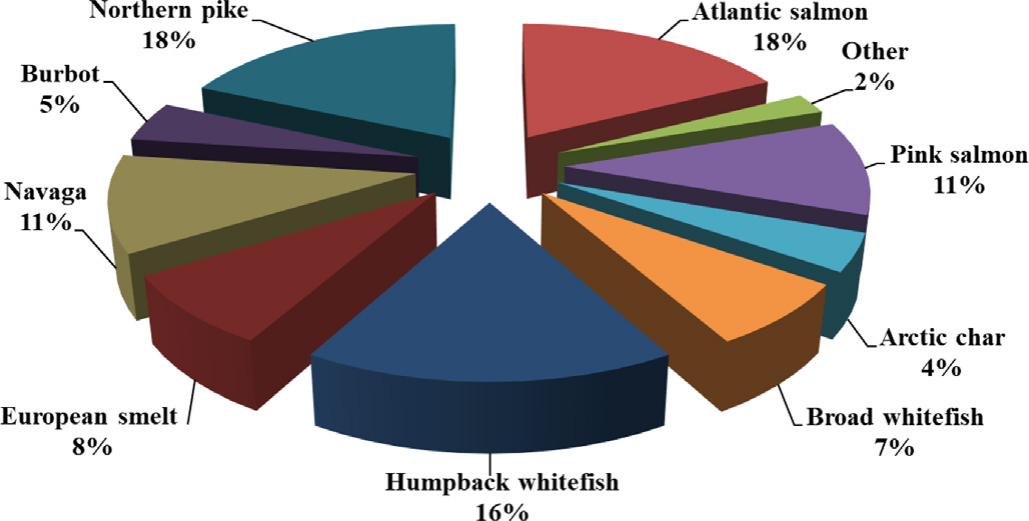
Pie-chart of the relative contributions of various species to the total fish consumption by indigenous Nenets.

Samples were collected within the Russian Arctic and sub-Arctic territories and the locations are indicated in Fig. 1. Fish species were selected with the guidance of a food-intake questionnaire administered during May 2017 to July 2018. Details about the average quantities of fish species consumed based on the questionnaire results are summarized in Table 1, while the relative contributions of various fish species to the total consumption are provided in a pie-chart format in Fig. 2. The raw data used to generate Table 1 and Fig. 2 are provided as Supplementary Material, as well as an English template of the questionnaire in Russian used. The raw elemental data measured in fish and examined in our recent article [1] are tabulated in Table 2. As these are to be updated later and due to the extent of the data, a Mendeley Data repository was created [2]. The data set will remain publicly available to local populations and authorities/agencies and is to be complemented by future field and analytical activities. It includes the following information: the age and weight of the fish, sampling dates, geographic coordinates and concentrations of Hg, As, Se, Cd, Pb, Co, Ni, Cu and Zn measured in muscle tissues. The moisture content of each sample was determined during the freeze-drying step and this permitted the expression of the elemental concentrations in μg/kg or mg/kg wet-weight (ww). Table 2 also features data for the fish species that were not included in the companion paper due to the small number of fish samples.

**Table 1:** Average consumption of fish species (kg/year) according to the questionnaires results (n = 150).

Fish spicie	Average consumption, kg/year
Atlantic salmon	10.0
Pink salmon	6.0
Arctic char	2.4
Broad whitefish	4.1
Humpback whitefish	9.4
European smelt	4.6
Navaga	6.2
Burbot	2.6
Northern pike	10.4
Other	1.3

**Table 2:** Geographic coordinates of the fishing sites and the elemental concentrations (wet wt) observed.

Fish specie	Sample name	Sampling date	Sampling site name	Age, years	Weight, kg	Hg	As	Se	Cd	Pb	Co	Ni	Cu	Zn
						μg/kg								mg/kg
Arctic char *(Salvelinus alpinus)*	94F	June 18, 2018	Indiga	6	0.90	20.9	2230	487	0.29	9.78	3.20	20.9	468	6.81
	96F			6.5	1.04	16.9	3690	405	0.54	5.84	3.71	18.9	480	6.31
	97F			5.5	0.82	14.3	2310	468	0.58	3.87	3.97	22.1	484	5.48
	98F			3.5	0.57	26.5	2040	423	0.84	8.90	3.08	22.8	390	3.76
	99F			3	0.47	20.3	3370	433	1.76	6.14	3.11	21.5	458	5.77
	100F			4.5	0.71	9.64	3760	528	0.28	3.79	2.92	20.8	517	5.46
	101F			3.5	0.59	16.4	1940	440	0.49	5.82	2.36	45.8	454	4.83
	102F			5.5	0.89	12.0	2750	551	0.22	7.31	2.87	23.1	445	5.36
	103F			4.5	0.73	12.6	2620	435	0.38	2.81	2.55	15.5	344	4.14
	104F			3.5	0.59	22.9	2010	404	0.33	0.99	2.34	9.40	394	3.99
	105F			2.5	0.25	27.6	2590	503	0.18	2.66	2.92	12.9	436	5.54
Pink salmon *(Oncorhynchus gorbuscha)*	82F	June 20, 2018	Indiga	1+	1.06	22.3	1000	444	7.11	8.65	2.59	9.90	647	8.04
	83F			1+	1.10	15.4	605	459	4.10	8.99	2.81	20.6	602	7.46
	84F			1+	0.92	16.3	875	454	6.11	11.9	2.02	13.2	591	8.35
	85F			1+	0.91	21.4	610	509	9.77	5.96	2.48	15.9	585	7.59
	86F			1+	1.64	16.5	843	581	2.17	13.4	2.60	13.1	452	6.64
	87F			1+	1.38	17.9	1080	653	8.98	3.92	2.34	15.3	486	8.30
	88F			1+	0.81	22.4	714	636	7.34	9.08	3.14	16.3	525	7.34
	89F			1+	0.84	16.4	513	521	4.05	7.47	2.36	18.4	484	7.17
	90F			1+	0.86	17.9	875	488	5.87	8.61	2.50	16.7	438	5.76
	91F			1+	1.21	19.5	1050	537	8.68	0.45	2.82	23.1	557	7.19
	92F			1+	0.91	17.2	783	539	6.42	18.7	3.07	19.5	549	6.59
	93F			1+	1.21	21.1	786	565	8.19	6.26	2.86	22.8	566	7.03
Navaga *(Eleginus nawaga)*	72F	March 17, 2018	Indiga	5.5	0.25	54.9	22900	412	0.94	6.26	9.80	20.4	507	12.5
	73F			4.5	0.22	74.2	59900	603	4.14	7.47	6.30	15.0	1020	8.48
	74F			5	0.28	35.1	10300	442	1.51	9.52	5.48	20.2	535	9.84
	75F			3.5	0.14	28.7	14800	660	2.15	6.77	6.83	22.3	668	16.0
	76F			4	0.14	59.8	46300	682	2.15	10.7	13.9	26.5	850	13.3
	77F			4.5	0.22	56.6	38400	633	1.56	7.99	8.05	24.0	732	10.5
	78F			4	0.20	117	45700	471	2.64	5.75	10.3	30.0	668	16.5
	79F			3	0.12	73.6	65500	599	2.73	5.41	14.0	27.7	473	11.5
	80F			6.5	0.38	77.2	25500	518	2.01	9.14	8.67	34.9	1100	18.4
	81F			5	0.25	57.7	20800	626	1.45	24.4	14.1	27.9	677	13.0
Humpback whitefish *(Coregonus pidschian)*	106F	June 18, 2018	Indiga	5.5	0.42	44.1	1240	396	0.90	1.54	26.7	17.3	254	6.77
	107F			6.5	0.42	49.7	1050	347	1.31	1.36	14.7	15.0	164	5.56
	108F			5	0.41	49.2	579	352	1.05	1.30	32.9	21.6	224	6.18
	109F			6	0.39	49.5	419	344	1.82	0.68	19.4	13.8	187	5.61
	110F			6.5	0.43	42.8	333	328	0.52	1.02	18.3	18.6	171	6.55
	111F			6.5	0.38	29.0	524	315	0.56	1.98	13.7	17.5	176	5.44
	112F			8	0.56	68.1	354	281	2.32	2.96	11.9	22.4	240	5.91
	113F			8.5	0.45	45.9	1240	366	0.40	4.50	17.4	23.9	181	5.66
	114F			10	0.54	90.6	815	305	5.09	6.32	14.6	25.2	183	4.87
	115F			8	0.46	111	2360	334	3.57	1.86	7.75	21.4	149	5.53
	116F			8	0.43	33.2	574	358	0.96	1.26	31.3	24.7	198	5.67
	117F			7.5	0.40	49.6	1240	358	1.12	2.38	28.9	29.2	250	6.67
Northern pike *(Esox lucius)*	6F	July 01, 2017	Pechora 1	8.5	3.32	157	1640	215	1.06	11.8	2.45	40.2	193	4.56
	7F			8	3.97	248	1830	181	0.49	8.05	1.33	19.7	130	3.48
	8F			10.5	5.62	312	628	168	0.49	7.58	2.51	11.7	204	3.61
	9F			7.5	3.17	189	342	178	0.33	11.3	1.55	16.9	248	4.18
	10F			3.5	0.81	130	5380	156	0.11	5.78	1.49	12.2	153	4.01
	11F			5	0.97	160	157	157	1.24	14.3	2.77	30.3	187	5.39
Roach *(Rutilus rutilus)*	135F	July 31, 2018	Pechora 2	8	0.31	86.0	50.0	269	3.12	5.99	2.74	21.7	307	5.30
	136F			8.5	0.32	73.9	51.7	307	4.19	4.26	3.62	19.8	370	8.16
	137F			8.5	0.31	107	113	298	3.02	2.20	3.37	17.7	455	5.20
	138F			8	0.33	101	50.2	306	3.45	2.98	4.19	20.4	301	6.41
	139F			9	0.33	79.2	89.9	315	2.96	3.02	3.28	19.6	268	7.22
	140F			10	0.35	113	62.3	285	3.93	4.18	2.94	17.4	199	6.28
	141F			7.5	0.28	85.5	75.5	315	2.33	5.80	2.74	19.1	317	8.39
	142F			6.5	0.24	92.9	80.1	287	3.37	2.66	3.58	17.2	305	7.73
	143F			8	0.31	85.8	69.2	269	3.03	3.29	2.82	14.9	193	5.76
	144F			8.5	0.31	117	76.4	295	3.19	4.29	3.82	18.5	245	7.08
	62F	May 12, 2018	Indiga	9.5	0.30	65.3	63.3	192	2.28	4.34	2.77	28.2	366	6.29
	63F			9.5	0.32	96.6	86.8	284	3.39	0.45	2.67	15.2	280	7.50
	64F			9	0.26	95.3	131	256	3.68	1.05	2.57	17.3	236	6.32
	65F			12.5	0.38	87.7	99.8	309	2.43	1.17	2.40	17.2	241	6.75
	66F			10.5	0.28	107	118	371	3.85	18.3	4.62	32.1	623	7.65
	67F			8.5	0.32	77.2	87.8	344	2.78	5.26	3.64	22.4	493	8.43
	68F			13	0.32	117	58.1	383	2.73	4.99	4.42	29.8	406	9.45
	69F			10	0.33	89.5	108	403	3.11	12.1	7.74	21.4	344	7.35
	70F			11.5	0.30	121	104	416	6.33	14.3	5.14	25.0	287	7.18
	71F			11	0.30	129	43.9	420	4.13	4.40	2.36	21.7	224	4.78
Inconnu *(Stenodus leucichthys nelma)*	54F	April 10, 2018	Pechora 1	7.0	1.50	120	1360	176	0.07	0.62	3.59	12.0	179	4.79
	55F	March 20, 2018		5.0	0.75	54.0	<LOQ	76.4	<LOQ	<LOQ	1.89	9.82	329	4.95
	26F	July 01, 2017		11.5	2.29	<LOQ	<LOQ	357	<LOQ	1.23	10.1	16.7	334	5.08
	41F			5.5	0.48	<LOQ	742	195	0.10	1.60	7.65	22.6	431	6.69
	31F			5.5	0.42	114	116	183	0.49	1.11	12.3	23.4	416	6.36
	25F			12.5	1.24	<LOQ	<LOQ	259	0.12	1.16	31.4	15.6	333	4.79
Arctic Flounder (*Liposetta glacialis*)	123F	June 18, 2018	Indiga	7	0.14	12.3	13700	2330	0.50	16.1	35.5	87.1	536	6.08
	124F			6.5	0.12	21.3	14700	2030	1.78	233	48.2	103	828	8.07
	125F			5.5	0.13	15.3	13400	2240	0.80	8.89	43.8	95.4	481	6.64
	56F	February 20, 2018	Pechora 1	5	0.20	16.4	274	475	6.01	6.76	26.0	274	756	26.0
Grayling *(Thymallus thymallus)*	52F	March 15, 2018	Kuloi 1	8	0.61	106	38.4	692	0.28	<LOQ	13.8	23.5	732	6.32
	58F	April 15, 2018	Pechora 1	6.5	0.50	42.6	62.8	128	0.39	2.72	53.1	21.0	631	6.49
	59F			7	0.45	55.2	53.0	116	0.55	15.9	48.2	33.8	777	8.21
Burbot *(Lota lota)*	53F	February 20, 2018	Pechora 1	6.5	0.75	36.4	9520	323	0.68	<LOQ	10.2	15.3	486	9.02
	57F			5.5	0.90	33.3	12800	360	0.37	2.26	25.1	20.8	472	8.76
Peled *(Coregonus peled)*	17F	July 01, 2017	Pechora 1	1.5	0.32	98.4	22700	357	<LOQ	<LOQ	10.1	16.7	334	5.08
	27F			1.5	0.35	5.34	742	195	0.10	<LOQ	7.65	22.6	431	6.68
	28F			4.5	0.36	<LOQ	1840	343	<LOQ	16.9	15.0	24.3	369	6.42
Broad whitefish *(Coregonus nasus)*	23F	July 01, 2017	Pechora 1	13	1.71	<LOQ	<LOQ	330	<LOQ	<LOQ	9.10	12.2	224	5.20
	24F			11.5	1.59	22.8	<LOQ	440	0.15	<LOQ	8.88	19.4	253	4.43
European perch *(Perca fluviatilis)*	3F	May 29, 2018	Pechora 3	9.5	0.52	194	<LOQ	301	0.18	<LOQ	6.76	17.0	411	6.01
	4F			10	0.55	243	<LOQ	377	0.10	<LOQ	8.78	26.7	533	7.23
	5F			8.5	0.43	214	<LOQ	322	0.42	<LOQ	8.03	37.8	433	6.44
Atlantic salmon *(Salmo salar)*	1F	June 17, 2017	Pechora 1	n/d	n/d	<LOQ	8790	498	0.32	2.57	9.18	22.1	1290	6.86
	2F			n/d	n/d	<LOQ	1990	283	<LOQ	2.69	<LOQ	33.7	714	5.51
	35F	July 01, 2017		n/d	0.31	<LOQ	1220	539	0.49	2.49	11.4	10.8	1200	7.59
	46F	March 23, 2018	Kuloi 2	n/d	n/d	<LOQ	2140	484	2.31	4.20	10.3	32.9	1000	5.90

## 2 Experimental design, materials, and methods

### 2.1 Study area description

Three villages (Krasnoe, Nelmin-Nos and Indiga) with a combined total population of 3059 and of whom ∼65% identified themselves as Nenets constituted the study sites. These villages are located on the shore of the Barents Sea, and the latter constitutes their primary food source. Based on our questionnaire information, the average total fish consumption by the study population was approximately 57 kg/year. Generally speaking, fish are caught predominantly at near-shore locations and by the indigenous people themselves.

### 2.2 Sample collection, preparation and analysis

Fish samples collected for analysis were bought from local fishermen on the same day they were caught. Sample collection spanned the period May 2017 to July 2018. The sampling sites for the fish species analysed are depicted in Fig. 1 and are also specified in Table 2. The names and geographic locations of the sampling sites and subsites are indicated in the Specifications Table above; see the project's data repository for additional information [2]. The coordinates for the sampling collection sites were noted and provided by the fisherman. The most common fish species consumed were identified by the responses to the mentioned questionnaire. The participants (n = 150) were drawn from the villages of Krasnoe, Indiga and Nelmin-Nos and the mentioned questionnaire was administered by the researcher to obtain pertinent information about what type of fish species and quantities they consumed every month. The data on the amount and type of fish commonly eaten by the participants are presented in a pie-chart in Fig. 2. To calculate the annual average fish consumption (wet-weight) for each participant interviewed, the total monthly intake by the entire study cohort was first calculated. The latter was subsequently divided by the number of participants and then multiplied by 12.

For the analyses, 0.25 g of homogenized/freeze-dried fish muscle samples were treated with 5 ml concentrated nitric acid in 50 ml PP tubes, and subsequently were diluted to 25 ml and analysed by ICP-MS. The limit of quantification for the elements were estimated as: Hg (1.0); As (35); Se (18); Cd (0.030); Pb (0.30); Co (1.0); Ni (1.1); Cu (4.0) in μg/kg, and Zn (0.020) mg/kg of wet-weight. Full details of the sample preparation procedures, fish age determination and ICP-MS analyses have been provided in the companion paper [1].
