# Supplementary material for: Concentration dataset for 4 essential and 5 non-essential elements in fish collected in Arctic and sub-Arctic territories of the Nenets Autonomous and Arkhangelsk regions of Russia
Source: Data Brief. 2019 Oct 18;27:104631. doi: 10.1016/j.dib.2019.104631 (PMC6838401; doi:10.1016/j.dib.2019.104631)
Supplement: Multimedia component 1 [file mmc1.docx]

**АНКЕТА ДЛЯ ОПРОСА НАСЕЛЕНИЯ**

**(QUESTIONNAIRE FOR POPULATION)**

*(Date of the interview)* Дата интервью:_______________________________________ день/месяц/год (day/month/year)

*(Full name of questioner)* Интервьюер: _______________________________________

**A. ОБЩИЕ Сведения**

**(COMMON INFORMATION)**

(Last name) Фамилия: _________________________________________________________________

(First Name) Имя: _____________________________________________________________________

(Patronymic) Отчество: _______________________________________________________________

(Gender) Пол: _______________

(Residence location: republic/region/district) Место проживания: республика/край/округ/область

____________________________________________________________________________

(District) район

(Town/ settlement/ village) город/поселок/село_____________________________________________

A1. (Date of birth) Дата рождения:

A2. (Current weight) Ваш вес в настоящее время:

A3. (Height) Ваш рост:

A4. (Nationality) Ваша национальность:

A5. (What type of home do you live in?) В жилище какого типа Вы живете?

(If you are living in flat, please specify the number of rooms) (Если квартира, то указать количество комнат)

(Summer residence) Летом:

(Winter residence) Зимой:

A6. (Family status) Семейное положение

(Married) Женат/Замужем (Divorced) Разведен(а) (Widow(er)) Вдовец(а) (Single) Одинок(а) (Unregistered marriage) Гражданский брак

A7. (Number of people living with you (including you)) Число совместно проживающих с Вами лиц (включая Вас)

(Women older than 15) Женщин 15 лет и старше

(Men older than 15) Мужчин 15 лет и старше

(Children younger than 15) Детей младше 15 лет

A8. (Do you have a permanent job now?) Есть ли у Вас в настоящее время постоянная работа?

(Yes) Да (No) Нет

(If yes, specify: seasonal or permanent) Если да, то какая (сезонная или непрерывная)?

(Place of Work) Место работы__________________________________________________________

(Profession) Профессия________________________________________________________________

A9. (Your education) Ваше образование?

- Не имею (none)
- Начальное (elementary)
- Неполное среднее (incomplete secondary education)
- Среднее (secondary education)
- Среднеспециальное (specialized secondary education)
- Высшее (higher education)

**Б. ОБРАЗ ЖИЗНИ**

**(LIFESTYLE)**

Б1. (Do you consume local foods?) Употребляете ли Вы в пищу продукты местного происхождения?

(Yes) Да____________ (No) Нет_____________

(If yes, complete Table 1) Если да, то заполните следующую таблицу 1:

(If no, go to Table 2) Если нет, то переходите к таблице 2.

**(Table 1)** Таблица 1.

| Наименование продукта питания  (Name of the product) | **Частота потребления местных продуктов питания**  (**Frequency of local food consumption**)  (включая всевозможные способы приготовления пищи – сырая, вареная, жареная, копченая, соленая, вяленая, маринованная …)  (including every type of food & cooking method – fresh, boiled, fried,  smoked, salted, dried, pickled, … | | | | | | |
| --- | --- | --- | --- | --- | --- | --- | --- |
|  | Количество пищи на один прием, (**граммы сырого веса**)  (Number of food items per meal; **in grams of wet weight**) | Еже-дневно  (Every day) | 1-3 раза в неделю  (1-3 times per week) | 2-3 раза в месяц  (2-3 times per month) | 1 раз в месяц или реже  (1 or less times per month) | (Fishing, hunting & harvesting) Промысел (ловля, охота, сбор) | |
|  |  |  |  |  |  | Place Место | Date (month) Время (месяц года) |
| **МЛЕКОПИТАЮЩИЕ (включая солонину и вяленое мясо) (MAMMALS) (All types of cooked meat)** | | | | | | | |
| (Seal meat) Мясо тюленя |  |  |  |  |  |  |  |
| (Seal fat) Жир тюленя |  |  |  |  |  |  |  |
| (Walrus meat) Мясо моржа |  |  |  |  |  |  |  |
| (Walrus fat) Жир моржа |  |  |  |  |  |  |  |
| (Whale meat) Мясо кита |  |  |  |  |  |  |  |
| (Whale fat) Жир кита |  |  |  |  |  |  |  |
| (Polar bear meat) Мясо белого медведя |  |  |  |  |  |  |  |
| (Polar bear fat) Мясо бурого медведя |  |  |  |  |  |  |  |
| (Venison) Оленина |  |  |  |  |  |  |  |
| (Elk) Лосятина |  |  |  |  |  |  |  |
| (Hare) Зайчатина |  |  |  |  |  |  |  |
| (Wild boar meat) Мясо кабана |  |  |  |  |  |  |  |
| (Other) Мясо другого зверя:  …………………….  …………………………………………………… |  |  |  |  |  |  |  |
| **ПТИЦА (BIRDS)** | | | | | | | |
| (Goose) Гусь |  |  |  |  |  |  |  |
| (Duck) Утка |  |  |  |  |  |  |  |
| (Partridge) Куропатка |  |  |  |  |  |  |  |
| (Other) Другие виды дичи:  …………………….  ……………………………………………………………………………… |  |  |  |  |  |  |  |

| **РЫБА (включая строганину, вареную, жареную, соленую, вяленую …)**  **(FISH) (All fried/cooked types)** | | | | | | | |
| --- | --- | --- | --- | --- | --- | --- | --- |
| (Trout) Форель |  |  |  |  |  |  |  |
| (Atlantic salmon) Семга |  |  |  |  |  |  |  |
| (Chum Salmon) Кета |  |  |  |  |  |  |  |
| (Pink salmon) Горбуша |  |  |  |  |  |  |  |
| (Arctic char) Голец |  |  |  |  |  |  |  |
| (Broad whitefish) Чир |  |  |  |  |  |  |  |
| (Humpback Whitefish) Сиг |  |  |  |  |  |  |  |
| (European Smelt) Корюшка |  |  |  |  |  |  |  |
| (Navaga) Навага |  |  |  |  |  |  |  |
| (Burbot) Налим |  |  |  |  |  |  |  |
| (Northern Pike) Щука |  |  |  |  |  |  |  |
| (Other) Другие виды рыбы:  …………………………………………………………………………………………………………. |  |  |  |  |  |  |  |
| (Crabs and shrimps) Крабы, креветки |  |  |  |  |  |  |  |
| (Crayfish) Раки |  |  |  |  |  |  |  |
| **ЯГОДЫ (включая варенье) (BERRIES) (Including jam)** | | | | | | | |
| (Cowberry) Брусника |  |  |  |  |  |  |  |
| (Cranberry) Клюква |  |  |  |  |  |  |  |
| (Blueberry) Черника |  |  |  |  |  |  |  |
| (Cloudberry) Морошка |  |  |  |  |  |  |  |
| (Raspberry) Малина |  |  |  |  |  |  |  |
| (Other) Другие виды ягод:  ……………………………………………………………………………… |  |  |  |  |  |  |  |
| **ГРИБЫ (включая жареные, сушеные для супов, соленые, маринованные…) (MUSHROOMS) (All types of cooked mushrooms)** | | | | | | | |
| (All mushroom species) Все виды грибов |  |  |  |  |  |  |  |
| **ОВОЩИ МЕСТНОГО ПРОИСХОЖДЕНИЯ, в т.ч. выращиваемые вами (включая домашние заготовки – соленые, консервированные, маринованные овощи) (VEGETABLES OF LOCAL ORIGIN INCLUDING THOSE YOU GROW) (including preserved vegetables)** | | | | | | | |
| (Potatoes) Картофель |  |  |  |  |  |  |  |
| (Carrot) Морковь |  |  |  |  |  |  |  |
| (Beet) Свекла |  |  |  |  |  |  |  |
| (Onion) Лук |  |  |  |  |  |  |  |
| (Cabbage) Капуста |  |  |  |  |  |  |  |
| (Lettuce) Салат |  |  |  |  |  |  |  |
| (Radish) Редис |  |  |  |  |  |  |  |
| (Tomatoes) Помидоры |  |  |  |  |  |  |  |
| (Cucumbers) Огурцы |  |  |  |  |  |  |  |
| (Other) Другие овощи:  ……………………………………………………………………………… |  |  |  |  |  |  |  |

(Table 2) Таблица 2.

| Наименование продукта питания  (Name of the product) | **Частота потребления привозных продуктов питания**  (C**onsumption Frequency of Imported foods**)  (включая всевозможные способы приготовления пищи – сырая, вареная, жареная, копченая, соленая, вяленая, маринованная …)  (including every type of cooking method – fresh, boiled, fried,  smoked, salted, dried, pickled, … | | | | | | |
| --- | --- | --- | --- | --- | --- | --- | --- |
|  | Количество пищи на один прием, (**граммы сырого веса**)  Number of food items per meal; (**grams wet- weight**) | Еже-дневно  (Every day) | 1-3 раза в неделю  (1-3 times per week) | 2-3 раза в месяц  (2-3 times per month) | 1 раз в месяц или реже  (1 or less times per month) | Место и дата покупки  (Origen and date) | |
|  |  |  |  |  |  | Место  (Place) | Дата  (Date) |
| **МЯСОПРОДУКТЫ (MEAT)** | | | | | | | |
| (Beef) Говядина |  |  |  |  |  |  |  |
| (Pork) Свинина |  |  |  |  |  |  |  |
| (Mutton) Баранина |  |  |  |  |  |  |  |
| (Chicken) Кура |  |  |  |  |  |  |  |
| (Other) Другие виды мяса и птицы:  ………………………………………………………………………………. |  |  |  |  |  |  |  |
| (Canned beef) Тушенка говяжья (консервы) |  |  |  |  |  |  |  |
| (Canned pork) Тушенка свиная (консервы) |  |  |  |  |  |  |  |
| (Dumplings) Пельмени |  |  |  |  |  |  |  |
| (Sausages) Сосиски, сардельки |  |  |  |  |  |  |  |
| (Boiled Sausage) Колбаса вареная |  |  |  |  |  |  |  |
| (Smoked Sausage) Колбаса копченая |  |  |  |  |  |  |  |
| (Ham and smoked meats) Ветчины и другие копчености |  |  |  |  |  |  |  |
| (Eggs) Яйца |  |  |  |  |  |  |  |
| **РЫБА (FISH)** | | | | | | | |
| (Cod) Треска |  |  |  |  |  |  |  |
| (Pollock) Минтай |  |  |  |  |  |  |  |
| (Hake) Хек |  |  |  |  |  |  |  |
| (Herring) Сельдь |  |  |  |  |  |  |  |
| (Mackerel) Скумбрия |  |  |  |  |  |  |  |
| (Halibut) Палтус |  |  |  |  |  |  |  |
| (Other) Другие виды рыбы:  ……………………………………………………………………………… |  |  |  |  |  |  |  |
| (Canned fish) Рыбные консервы:  ……………………………………………………………………………… |  |  |  |  |  |  |  |
| (Crabs, shrimps) Крабы, креветки |  |  |  |  |  |  |  |
| **ОВОЩИ (VEGETABLES)** | | | | | | | |
| (Potatoes) Картофель |  |  |  |  |  |  |  |
| (Carrot) Морковь |  |  |  |  |  |  |  |
| (Beet) Свекла |  |  |  |  |  |  |  |
| (Onion) Лук |  |  |  |  |  |  |  |
| (Cabbage) Капуста |  |  |  |  |  |  |  |
| (Lettuce ??) Салат |  |  |  |  |  |  |  |
| (Radish) Редис |  |  |  |  |  |  |  |
| (Tomatoes) Помидоры |  |  |  |  |  |  |  |
| (Cucumbers) Огурцы |  |  |  |  |  |  |  |
| (Other) Другие овощи:  ……………………………………………………………………………… |  |  |  |  |  |  |  |
| (Canned vegetables) Овощные консервы:  ……………………………………………………………………………….. |  |  |  |  |  |  |  |
| **ФРУКТЫ (FRUITS)** | | | | | | | |
| (Oranges) Апельсины |  |  |  |  |  |  |  |
| (Apples) Яблоки |  |  |  |  |  |  |  |
| (Other) Другие фрукты:  ……………………………………………………………………………… |  |  |  |  |  |  |  |
| **МОЛОЧНЫЕ ПРОДУКТЫ (Milk PRODUCTS)** | | | | | | | |
| (Milk) Молоко |  |  |  |  |  |  |  |
| (Dairy products) Кисломолочные продукты |  |  |  |  |  |  |  |
| (Butter) Масло сливочное |  |  |  |  |  |  |  |
| (Cheese) Сыр |  |  |  |  |  |  |  |
| (Cottage cheese) Творог |  |  |  |  |  |  |  |
| (Other) Другие молочные продукты:  …………………………………………………… |  |  |  |  |  |  |  |
| **БАКАЛЕЯ И НАПИТКИ (CEREAL PRODUCTS AND DRINKS)** | | | | | | | |
| (Cereals) Крупы |  |  |  |  |  |  |  |
| (Pasta) Макаронные изделия |  |  |  |  |  |  |  |
| (Rye bread) Ржаной хлеб |  |  |  |  |  |  |  |
| (Wheat bread) Пшеничный хлеб |  |  |  |  |  |  |  |
| (Sugar) Сахар |  |  |  |  |  |  |  |
| (Juices) Соки |  |  |  |  |  |  |  |
| (Lemonade)Лимонад |  |  |  |  |  |  |  |
| (Mineral water) Минеральная вода |  |  |  |  |  |  |  |
| (Bottled water) Вода очищенная |  |  |  |  |  |  |  |

Б2. (What are the sources of your drinking water?) Откуда Вы получаете питьевую воду:

| Источник воды  (Water source) | Потребление воды по месяцам (пометить галочками)  Water consumption every month (use checkmarks) | | | | | | | | | | | |
| --- | --- | --- | --- | --- | --- | --- | --- | --- | --- | --- | --- | --- |
|  | Янв  (Jan) | Фев  (Feb) | Мар  (March) | Апр  (Apr) | Май  (May) | Июн  (June) | Июл  (July) | Авг  (Aug) | Сен  (Sep) | Окт  (Oct) | Ноя  (Nov) | Дек  (Dec) |
| (Сentral water supply)  Водопровод |  |  |  |  |  |  |  |  |  |  |  |  |
| (Water pipes) Колонка |  |  |  |  |  |  |  |  |  |  |  |  |
| (Well) Колодец |  |  |  |  |  |  |  |  |  |  |  |  |
| (Water reservoir) (Creek, river, lake) Водоем (ручей, река, озеро) |  |  |  |  |  |  |  |  |  |  |  |  |
| (Melted water) (snow, ice) Талая вода (снег, лед) |  |  |  |  |  |  |  |  |  |  |  |  |

Б3. (Do you smoke?) Курите ли Вы? (Yes) Да________ (No) Нет____________

(If yes how many years?) Если да, то сколько лет подряд Вы курите?……………………….

(What age did you start smoking?) В каком возрасте Вы начали регулярно курить?…………………..

(What do you smoke?) (cigarettes with filter, without filter, smoking pipe, cigars) Что Вы курите (сигареты с фильтром, папиросы, трубку, сигары)?……………………………….

(How many times per day do you smoke?) Сколько раз в день Вы курите?……………………………………………

(If you do not smoke, did you smoke previously?) Если не курите, то курили ли Вы раньше? (Yes) Да________ (No) Нет____________

(What age did you start to smoke?) В каком возрасте Вы начали регулярно курить?…………………..

(What age did you finish smoking?) В каком возрасте Вы совсем бросили курить?…………………….

(How many persons (without you) smoke in your house?) Сколько человек (кроме Вас) курят в Вашем доме?………………………………..

Б4. (Do you use chemicals for insect control? (e.g. Cockroaches, Flies, Mosquitoes, bedbugs) Применяете ли Вы дома химикаты для борьбы с насекомыми (тараканами, мухами, клопами)

(Yes) Да ________ (No) Нет ________

(Chemicals for insect control in your garden?) химикаты для борьбы с насекомыми на своем огороде (Yes) Да ________ (No) Нет ________

(Chemicals for insect control in your work place) (for example for animal treatment) химикаты для борьбы с насекомыми на работе (напр., для обработки животных)

(Yes) Да ________ (No) Нет ________

(Chemicals to control rodents) химикаты для борьбы с грызунами (Yes) Да _____ (No) Нет __________

(Which chemicals do you use?) Какие именно химикаты Вы используете? ______________________________________

(How often do you use chemicals times per:) Как часто Вы используете химикаты - сколько раз:

(week) в неделю ………………

(month) в месяц………………..

(year) в год …………………..

(Where do you store chemicals?) Где Вы храните химикаты?

Б5. (Do you catch fish?) Ловите ли Вы рыбу? (Yes) Да _______ (No) Нет ___________

(If yes do you use sinkers made of lead?) Если да, то используете ли Вы свинцовые грузила в процессе ловли?

(Yes) Да _______ (No)Нет ___________

(Do you melt (produce) sinkers yourself?) Льёте ли Вы грузила самостоятельно?

(Yes) Да _______ (No) Нет ___________

Б6. (Do you hunt?) Охотитесь ли Вы? (Yes) Да _______ (No)Нет ___________

(If yes, which types of ammunition do you use?) Если да, то какого типа и торговых марок свинцовые пули и дробь Вы используете для охоты?

________________________________________________________________________________

________________________________________________________________________________

________________________________________________________________________________

(How many boxes of cartridges do you use per year?) Сколько коробок с патронами Вы используете в течение года? ___________________________

(How many days per year do you hunt?) Сколько дней в году Вы охотитесь?___________________________
